# Infant Massage and Quality of Early Mother–Infant Interactions: Are There Associations with Maternal Psychological Wellbeing, Marital Quality, and Social Support?

**DOI:** 10.3389/fpsyg.2016.02049

**Published:** 2017-01-17

**Authors:** Alessio Porreca, Micol Parolin, Giusy Bozza, Susanna Freato, Alessandra Simonelli

**Affiliations:** Department of Developmental and Social Psychology, University of PaduaPadua, Italy

**Keywords:** infant massage, mother–child interactions, child development, parenting, early relationships

## Abstract

Infant massage programs have proved to be effective in enhancing post-natal development of highly risk infants, such as preterm newborns and drug or HIV exposed children. Less studies have focused on the role of infant massage in supporting the co-construction of early adult–child relationships. In line with this lack of literature, the present paper reports on a pilot study aimed at investigating longitudinally the quality of mother–child interactions, with specific reference to emotional availability (EA), in a group of mother–child pairs involved in infant massage classes. Moreover, associations between mother–child EA, maternal wellbeing, marital adjustment, and social support were also investigated, with the hypothesis to find a link between low maternal distress, high couple satisfaction and high perceived support and interactions of better quality in the dyads. The study involved 20 mothers and their children, aged between 2 and 7 months, who participated to infant massage classes. The assessment took place at three stages: at the beginning of massage course, at the end of it and at 1-month follow-up. At the first stage of assessment self-report questionnaires were administered to examine the presence of maternal psychiatric symptoms (SCL-90-R), perceived social support (MSPSS), and marital adjustment (Dyadic Adjustment Scale); dyadic interactions were observed and rated with the Emotional Availability Scales (Biringen, 2008) at each stage of data collection. The results showed a significant improvement in the quality of mother–child interactions, between the first and the last evaluation, parallel to the unfolding of the massage program, highlighting a general increase in maternal and child’s EA. The presence of maternal psychological distress resulted associated with less optimal mother–child emotional exchanges, while the hypothesis regarding couple satisfaction and social support influence were not confirmed. These preliminary results, if replicated, seem to sustain the usefulness of infant massage and the importance of focusing on early mother–infant interactions.

## Theoretical Background

### Infant Massage

The practice of infant massage represents a simple but effective way to enhance and strengthen healthy social and emotional relationships between adults and children in early infancy; this is true both from a relational and a practical point of view. On the one hand, in fact, massaging a baby requires and intensifies a series of multimodal and interactive competencies (such as emotional expression, eye-contact, physical touch, vocal communication, and turn-taking) that usually characterize adult–child daily repertoires; in this sense, it represents a privileged moment to cultivate and strengthen the relationship. On the other hand, instead, it is cost-saving and no contraindications were reported up to now; thus, it can be used frequently and without risk, accompanying and supporting a process existing per se, which is the one of adult–infant bonding.

Together with the practices of breastfeeding, baby carrying, and co-sleeping, infant massage is part of a wider “caretaking-package” which involves a set of behaviors necessary to satisfy the child’s needs for contact, holding, communication, and feeding; these needs are simple and primitive but often unrecognized (Balsamo, 2007). A particular feature of these behaviors is that they specifically require tactile interactions with the infant and that they are able to convey a series of somatosensory messages about feelings, pressure, temperature, softness, or pain (Underdown, 2009). As already shown by the research on sensorial deprivation in early infancy, these experiences are fundamental for physical growth, neurological development and for the construction of healthy affective relationships (Robertson and Bowlby, 1952; Harlow and Zimmermann, 1958; Montagu, 1971; Blackwell, 2000; Kim et al., 2003).

From a neural-developmental point of view, touch is one of the first sensorial systems to be activated in the fetus during pregnancy, becoming, thus, one of the primary means of communication with the surrounding environment (Relier, 1994; Hernandez-Reif et al., 2007). After delivery, touch becomes an important channel of communication during mother–infant interactions (De Chateau, 1976; Tronick, 1995; Stack, 2004; Moszkowski and Stack, 2007; Jean and Stack, 2009; Stack et al., unpublished). It occurs more than the 55% of time and communicates security and tenderness, reducing infants’ distress and promoting emotional regulation (Stack and Muir, 1990, 1992; Weiss et al., 2000; Jean et al., 2004; Jean and Stack, 2009). Empirical studies have widely stressed that sensitive caregiving allows the baby not to feel overwhelmed, moderating or accelerating his/her emotions and intervening at a neuro-physiological level on the reactivity of the hypothalamic-pituitary-adrenocortical (HPA) system (Tronick, 1989; Nelson and Bosquet, 2000; Beebe and Lachmann, 2002). In this sense, early experiences of touch represent one of the means through which the adult works as an external regulator for the baby, helping the co-regulation of his/her behavioral and emotional states and consequently preventing him/her from experiencing developmental difficulties, such as sleep and feeding problems (De Chateau, 1976; Koniak-Griffin and Ludington-Hoe, 1988; Brazelton, 1990; Underdown, 2009).

Infant touching and infant massage are also part of a “high-” or “proximal-contact” model of caretaking (Stork, 1986; Balsamo, 2002), that promotes intense physical and psychological contact between mother and child, with the aim to hold the child and to protect him/her from dangers. From this point of view, and given the relational and developmental influences of early experiences of touch, infant massage appears a useful technique to support parenting by promoting sensitivity and enhancing the construction of healthy affective bonds between adults and children (Bozza, unpublished).

Research on infant massage as intervention has been applied especially to preterm infants. In this particular population the exposure to infant massage sessions resulted in an improvement in several health indexes, such as weight-gain, increases in length, head circumference, bone density, and body temperature (Scafidi et al., 1986; Scafidi et al., 1990; Kuhn et al., 1991; Wheeden et al., 1993; Moyer Mileur et al., 1995; Jinon, 1996; Dieter et al., 2001, 2003; Ke et al., 2001; Duan et al., 2002; Ferber et al., 2002; Liu, 2005; Lu et al., 2005; Na et al., 2005; Diego et al., 2008).

Moreover, massage therapy resulted also capable of modifying the distribution of sleep/awake states, favoring longer periods of active alertness and reducing excitability (Scafidi et al., 1986, 1990; Field et al., 1987, 2004; Wheeden et al., 1993; Dieter et al., 2003).

As far as it concerns other groups at high-risk, infant massage sessions have worked successfully on HIV- and drug-exposed newborns, leading to fewer medical complications, less irritability and increased weight, and to an improvement in the performance on social and emotional scales (Scafidi et al., 1990; Wheeden et al., 1993; Scafidi and Field, 1996; Dieter et al., 2001).

Although most non-at-risk infants will receive adequate sensitive handling, the administration of infant massage with regular frequency can represent an useful way to support parenting and to promote caregivers’ sensitive touch in early infancy (Underdown, 2009). Some authors emphasized the bidirectional effects of giving and receiving massage, supporting the use of the technique as a safe and cost-effective intervention for adult–child relationships (Feijò et al., 2006). Firstly, in fact, maternal resources can be enhanced by massage lessons through the promotion of a better knowledge of the infant’s needs and characteristics; secondly, the child’s features can be better modulated thanks to a more satisfying contact with the caregiver (Oswalt et al., 2009).

Massages can be given to children on a daily basis and they are economically saving when parents are enrolled as therapists (Field, 2002). The massage of infants promotes a general sense of wellbeing in the adult, helping the parent to feel close to the baby, and less fearful of touching and handling him/her. Moreover mothers who massaged their infants, both preterm and full term, reported less anxiety, less depressed mood, and improved mother–child interactions (Onozawa et al., 2001; Ferber et al., 2005; Feijò et al., 2006). Teaching parents to massage their infants, especially in risk conditions, often lowers anxiety levels related to the feelings of helplessness (Field, 2002). As far as it concerns the child, infant massage reduces and balances cortisol, epinephrine, and norepinephrine hormones which control stress levels (Acolet et al., 1993; Field et al., 1996). As a consequence, this practice could be considered as a facilitator of the normal development of these catecholamines that characterize early stages of life (Kuhn et al., 1991).

Promoting infant massage lessons may represent an ideal way to support parenting and to support early emotional and social relationships between adults and children, useful both for high- and low-risk groups (Underdown, 2009). This technique offers a perspective which is simple but very useful, since it combines a behavioral intervention (i.e., the physical manipulation of the baby) with a profoundly emotional dimension (i.e., the close psychological and emotional proximity that massage enhances). Moreover, participating to massage classes constitutes an opportunity to meet other adults that are living the same experience, to share and compare different points of view, to create new bonds (Adamson, 1996).

### Parenting and Early Interactions from a Multi-Determined Point of View

Parenting encompasses a broad range of nurturing and care-taking actions performed by caregivers toward the child. The actual behaviors that parents provide are among the most salient aspects of parenting, since the most of the experience of infants stems directly from interactions with caregivers occurring daily (Bornstein, 2002). Recently, increasing attention has been given to the emotional features that accompany parenting routines; more specifically, an important contribution to the investigation of parenting has been given by the theoretical frame of Emotional Availability (EA), which has pointed out the importance to create an emotional connection and to be able to share a wide range of affects during caretaking behaviors (Biringen, 2008; Biringen and Easterbrooks, 2012). As pointed out above, infant massage can be considered as a component of care-taking behaviors, that offers a valuable interactive context to parents, who can involve in pleasant and rewarding exchanges with their babies. Thanks to Belsky’s work on parenting (1984), nowadays it is well acknowledged that parenting, in all its components, is a multi-determined construct, encompassing both contextual variables and baby’s and parents’ individual characteristics. Empirical evidence attests that some of these factors are of particular relevance, such as the quality of marital relationship, maternal psychological wellbeing, and perceived social support.

The transition to parenthood is an event of the family life cycle that asks the couple to face potentially stressful changes and challenges (Belsky and Rovine, 1990; Cowan and Cowan, 2000) both at an inner and behavioral level (Cowan and Hetherington, 2001), in order to meet child’s need and develop parenting competences. In these terms, it is a critical period for marital satisfaction, which goes through a small but reliable decline, persisting at least until the preschool age as reported by several studies on dyadic satisfaction (Belsky et al., 1983; Terry et al., 1991; Favez et al., 2012; Kohn et al., 2012; Trillingsgaard et al., 2014). Women seem to experience a steeper and more precipitous drop in marital quality (Shapiro et al., 2000; Twenge et al., 2003; Bower et al., 2013), especially when they are scarcely satisfied with the division of labor and they manage a greater amount of childcare activities (Belsky, 1986; Gjerdingen and Center, 2005; Dew and Wilcox, 2011; Chong and Mickelson, 2013). However, other studies indicate that mothers and fathers share similar perception of post-birth relationship functioning (Lawrence et al., 2008; Doss et al., 2009; Jackson et al., 2014).

The transition toward parenthood is a very critical and stressful stage that may lead to serious psychological distress symptoms in pregnant women and women that recently gave birth to their offsprings, ranging from 4.8% (Glasheen et al., 2015) to 19% (Czarnocka and Slade, 2000; Saurel-Cubizolles et al., 2007; Gavin et al., 2011). Maternal psychological distress appears to be enduring (Horwitz et al., 2007) and, given that the early postpartum months are especially important for the establishment of a satisfactory dyadic relationship and for infant development (Hay and Kumar, 1995; Murray et al., 2015), it may negatively affect child outcomes (Goodman et al., 2011), mother–infant interactions (Singer et al., 2003), conjugal and family relationships (Whisman, 2001; Sutter-Dallay, 2006). Some studies highlight that marital conflict shows the most significant association with maternal distress in women who have recently given birth (Stemp et al., 1986).

Depression represents one of the most frequent distress conditions in the context of the transition to motherhood; review and meta-analytic studies have demonstrated that depression is linked to a range of adverse behavioral and emotional outcomes for the child, in terms of psychopathology and negative affects and behaviors, especially for younger children (Goodman, 2007; Goodman et al., 2011). Maternal depression is linked with impaired mother–infant interactive patterns since early age (i.e., 4 months) in both partners, especially concerning self-and interactive contingency (Beebe et al., 2012; Murray et al., 2015); depressed mothers are described by Weinberg and Tronick (1997) as intrusive or withdrawn. Notably, improving maternal depression does not imply per se an enhancement of the quality of mother–infant interaction, indicating the importance of targeting not merely mother’s depression and adopting instead a broader approach (Forman et al., 2007). A pilot study (Onozawa et al., 2001) reports that depressed mothers beneficiate from attending a massage class, attenuating depression symptoms and learning to interact better with their babies, leading in turn to an improvement in dyadic interaction (maternal attitudes and behaviors toward the child and infants’ responses), more than mothers in a support group did. Another study (O’Higgins et al., 2008) attests a clinically significant reduction in depression for the majority of mothers, even though scores remained high.

In a wider perspective, parenting and child development inextricably take place in the context of social relationships. Social support can be defined as the amount of advice received and personal needs fulfilled through the presence and interaction with significant others, within or outside the family, such as partners, relatives, or friends (Kaplan et al., 1977). Social support has been widely linked to individual wellbeing and positive mental health at an individual level (Mitchell and Trickett, 1980); in particular, women are more likely to give social support, draw on socially supportive networks in times of stress and, more importantly, benefit from it (Taylor, 2011).

Moreover, according to Belsky’s (1984) model, social support is a key determinant of parenting quality; it plays a role (as mediator, moderator or as a direct influence), in influencing parent–child interactions (Jennings et al., 1991), attitudes about parenting, parenting behaviors and, in turn, child outcomes (Feiring et al., 1987; Melson et al., 1993; Bender and Losel, 1998). Pregnant women that can rely on others for support show better outcomes for stress, depression and anxiety (Glazier et al., 2004). As regards mothers, the advantages of social support, especially if it is provided from one’s own mother, can be detected in the domains mental health, reducing the risk of postpartum depression, and of marital satisfaction (Findler et al., 2008); in fact, social support is important for the couple too, being associated to partners’ cohesion and intimacy (Cutrona, 1996). Nevertheless, support to mothers peaks in the first weeks after childbirth, but it appears to decrease in the following months (Pinelli, 2000; Rowe and Jones, 2010). If social support is typically considered as a positive influence on parenting, it is also important to note that it can exert an adverse influence, in terms of stressful or non-supportive relationships that lead to decreased wellbeing (Ingersoll-Dayton et al., 1997).

Interestingly, Herwig et al. (2004) have simultaneously considered maternal parenting and depression, couple satisfaction and social support in their associations with child outcomes, reporting the predictive role of maternal parenting and couple satisfaction and the indirect influence of depression and social support on child development.

## Aims and Hypothesis

To our knowledge, to date no studies have examined the quality of mother–infant interactions in the context of infant massage through the application of the Emotional Availability Scales (EAS; Biringen, 2008), nor have adopted a longitudinal approach. The present preliminary research aimed to investigate mother–child EA during infant massage classes. EA refers to the capacity of a dyad to share an emotionally healthy relationship and has been widely used in research to assess the quality of parenting and of adult–child relationships. Moreover, we adopted a longitudinal perspective, in order to verify whether EA changed or remained stable during the unfolding of infant massage lessons. Finally, according to the extant literature that highlights the role of multiple factors in shaping the quality of early parenting practices, we investigated whether EA was associated to mothers’ perception of couple adjustment, social support and psychological wellbeing. An increase in dyadic EA was expected and we hypothesized that more optimal adult–child interactions would be associated with a lower degree of maternal psychological distress, and with a higher level of couple satisfaction and perceived social support.

## Materials and Methods

The present study adopted a descriptive and correlational design for providing preliminary data on the longitudinal investigation of mother–infant EA during massage classes.

### Participants^1^

The sample of the study was composed of 20 mother–child pairs selected from a larger group of participants enrolled in an infant massage course. The children’s (10 boys and 10 girls) age ranged from 2 to 7 months (*M* = 2.6, *SD* = 1.392). Sixty-five percent of the subjects could be assessed longitudinally. The remaining subjects (35%) were not tested longitudinally due to obstacles preventing mothers from attending the scheduled lessons. The mothers were all Caucasian; their age ranged from 27 to 38 years old (*M* = 31.75, *SD* = 2.751). Inclusion criteria for the study included: being primiparous, being subject to the first experience of infant massage, having attended all massage classes with the same conductor, absence of infant pathology.

Socio-demographic information regarding different domains such as level of education and occupation was collected through a self-report questionnaire. Twenty percent of the mothers reported to be an only child, while the others reported one (40%) or more (40%) siblings. Concerning education, the 10% of the group declared to have a middle school certificate, while the others reported to have an high school diploma (35%) or an academic degree (50%). As far as it regards work, 15% of the mothers reported to be unemployed, while the remaining declared to have a job in the working class (10%), as employees (45%) or in other forms (30%).

### Procedure and Instruments

**FIGURE 1:**
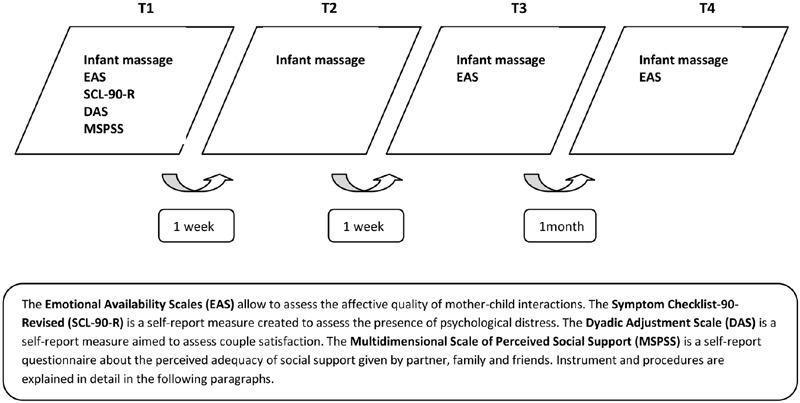
**Times of measurement, intervention, and variables assessed**.

Massage courses were presented during childbirth classes in different Venetian sanitary districts. Once enrolled the women were contacted after delivery and invited to participate to the course. Participation was free. The dyads were divided randomly in groups of 5, each of these represented a massage class. At the beginning of the course each mother was given a battery of self-report questionnaires to fill in at home, aimed at investigating socio-demographic information, maternal psychological well being, marital relationships and perceived social support (see the section Quality of Mother–Child Interactions during Massage Lessons). The participants were told that they would have been videotaped three times during the cycle of massage classes (during T1, T3, and T4). Informed consent was asked to both parents before videotaping the baby. **Figure 1** resumes the research design, and the variables assessed during the different times of measurement.

#### The Infant Massage Program

The dyads were offered a cycle of four weekly lessons on infant massage. Each encounter lasted about an hour and a half. Massage courses were provided by a trained psychotherapist. During the course each room was warm and quiet in its atmosphere. Cameras were located in different angles of the room at a distance that allowed to capture the most salient aspects of each dyad’s interaction without interfering with the appropriate atmosphere.

Every mother was told to bring a cushion big enough to contain the baby and some natural oil. Activities took place on the floor, sitting in circle on wickers, in front of their babies, holding cushions firm with their legs. This condition allowed the mothers to hold their babies and to enhance visual contact with them. The conductor sit in the center of the circle in order to be visible for all the participants, and reproduced on a doll the various techniques taught during each encounter. In this way, the conductor did not touch the babies but only showed the participants how to massage.

The first lesson (T1) concerned an initial introduction to the massage course and to the research project. After a brief presentation of the equipe, the mothers were given notions in order to experience in the best way possible the encounters: they were invited to pay attention and to follow their children’s needs, to wait for optimal moments before beginning massage sessions, to feed their babies or to let them sleep whether necessary, to calm them down as they were used to. After a brief moment of relaxation, the mothers were invited to undress their infants from waist down, in order to be able to massage their legs and their abdomen. Instructions focused on how to handle the baby and how to touch, on how to pay attention and to become aware of the baby’s signals. The mothers were lead through a group discussion on massage benefits and they were invited to repeat the procedure at home. The first encounter was video-recorded.

During the second lesson (T2) the conductor introduced another sequence of massage procedures, focusing on face, superior arts, and chest. Alternative positions to use massage were presented and parents were positively reinforced during their efforts. Moreover, the mothers were given the possibility to share their feelings about maternity with the group. The conductor stressed the fact that the massage represents a technique with a particular focus on mother–child relationship, a relationship that can be improved dedicating more time to practice at home and, in turn, to the relationship.

The third lesson (T3) concerned the repetition of the entire sequence applied to the frontal body, also adding some suggestions on how to approach the back during massage. Again, some space during this encounter was left to allow the mothers to talk about their experience of the postpartum period. A bibliography was suggested to acquire more knowledge regarding the themes emerged during the cycle of lessons. This encounter was video-recorded.

After a month the group met again for a so-called moment of reinforce and of review of the techniques previously learned. This session was videotaped (T4) and considered as a follow-up.

#### Quality of Mother–Child Interactions during Massage Lessons

The first (T1), the third (T3), and the fourth (T4) lessons were videotaped. For every dyad 20 min of mother–child interaction were recorded during each episode. The interactions were coded using the fourth version of the EAS (Biringen, 2008). The coding system is composed of six scales/dimensions, four for the adult (sensitivity, structuring, non-intrusiveness, and non-hostility) and two aimed at evaluating the child’s contribution (responsiveness, involvement of the adult).

*Adult sensitivity* refers to quality of adult affects, clarity of perceptions and appropriate responsiveness, awareness of timing, flexibility, variety and creativity during play, acceptance of the child, amount of interactions, and adequate resolution of conflicts.

*Adult structuring* concerns the use of proactive guidance, the success of attempts, the amount of guidance, the ability to set limits and to remain firm in the face of pressure, the use of both verbal and non-verbal suggestions and the ability to assume an adult role rather than a peer one.

*Adult non-intrusiveness* refers to the ability to follow the child’s lead, to the use of non-interruptive ports of entry into interaction, to the modest use of commands and directives, to the appropriateness of teaching and adult talking, to the absence of interferences and of child’s signals that indicate that the adult is perceived as intrusive.

*Adult non-hostility* refers to the lack of negativity in face or voice and to the lack of ridiculing or other disrespectful behaviors toward the child. A non-hostile adult does not threat to separate, is not frightening, maintains cool during challenging situations and does not use threats of hostile play themes during interactions.

*Child responsiveness* takes into account quality of child’s affects and organization of behaviors, the ability and the willingness to respond to the adult’s bids without anxiety or role reversal. It also considers positive physical positioning, concentration on task and the presence of avoidance or of over responsiveness and role reversal.

*Child involvement of the adult* concerns the use of simple and elaborative initiative to involve the adult, the affective use of the adult (rather than instrumental), the lack of negative/over involving behaviors, and the use of verbal and non-verbal channels.

Each EA dimension is given a global score on a 7-point scale, with higher ratings referring to more optimal features. Scores between 5 and 7 are considered adequate and index of a healthy relationship. Scores around 4 indicate inconsistency, (i.e., behaviors that are appropriate in some way but that are not fully healthy). Scores of 3 or below indicate less optimal interactions were problematic behaviors might arise (scores of 1 or 2). The coding refers to the global quality of the interaction observed rather than on specific behaviors. To get a more specific profile of the adult–child relationship, the EA assessment system provides the coders also a Clinical-Screener that allows to attribute each member of the dyad to one of four “zones” (according to the scores given to maternal sensitivity and child responsiveness), which represent four possible categories of EA: the Emotionally Available zone, the Complicated zone, the Detached zone and the Problematic zone. Mother–child interactions were coded by two independent raters previously trained on the EA coding system in order to reach satisfactory reliability with the Biringen’s lab. Inter-rater reliability was calculated on the 20% of the videos using ICCs which ranged from 0.80 to 0.95.

#### Self-report Measures

At the beginning of the massage course the mothers were given a battery of self-report questionnaires to fill in at home before the second lesson. The instruments aimed to assess socio-demographic information, maternal psychological wellbeing, marital relationships and perceived social support.

The *Symptom Checklist-90 Revised* (*SCL-90-R*; Derogatis, 1977; Italian version by Sarno et al., 2011) is a brief questionnaire designed to evaluate the presence of psychological distress and a range of psychopathological symptoms. It consists of 90 items and yields nine scores along primary symptom dimensions and three scores that refer to global distress indexes. The primary assessed symptom dimensions are somatization, obsessive-compulsive, interpersonal sensitivity, depression, anxiety, hostility, phobic anxiety, paranoid ideation, and psychoticism. The three global indexes refer to global psychological distress status (Global Severity Index – GSI), to the total number of symptoms reported (Positive Symptom Total – PST) and to the intensity of reported distress (Positive Symptom Distress Index – PSDI). According to T-scores, each item can be interpreted as below average scores, within the median range, above average scores and definitely above the average scores of the normative sample (thus indicating severe symptomatology).

The *Dyadic Adjustment Scale* (*DAS*; Spanier, 1976; Italian version by Gentili et al., 2002) is a multidimensional tool that allows to assess conjugal adjustment. The sum of the 32 items lead to four dimension of conjugal adjustment (Dyadic Consensus, Dyadic Satisfaction, Dyadic Cohesion, and Affective Expression) and to a global score (Total Adjustment Score) that represents the degree of conjugal adjustment perceived by the partners.

The *Multidimensional Scale on Perceived Social Support* (*MSPSS*; Zimet et al., 1988; Italian version by Prezza and Principato, 2002) is a self-report questionnaire about the perceived adequacy of support given by the partner, family and friends. The 12 items are given a score on a 7-point Likert Scale and their sum leads to three dimensions concerning support received by the family, by friends and by a significant other.

## Results

Data were analyzed using IBM SPSS statistics vers. 23. Firstly, descriptive statistics (frequencies, mean scores, and percentages) were examined. Secondly, non-parametric tests were applied; more specifically, we adopted the Friedman and Wilcoxon signed-rank and the Spearman’s Rho to test for any associations between the different instruments adopted in the research design.

### Preliminary Analysis

During preliminary analysis, Cronbach’s alpha coefficient was used to assess the reliability of the instruments. Descriptive statistics (average scores, frequencies, and percentages) were examined.

#### Mother–Child Interactions during Massage Lessons

The application of Cronbach’s alpha coefficient, to all the three periods considered, reported good reliability for EA maternal scales (0.85 ≤ α ≤ 0.89), for the EA child’s scales (0.73 ≤ α ≤ 0.80) and for all the six scales considered globally (0.87 ≤ α ≤ 0.90). **Tables 1** and **2** report average scores, standard deviations and the distribution of the dyads assessed through the EAS and the EA clinical screener. As it is possible to observe from **Table 1**, regarding the EA Scales, during T1 the dyads reported on average score 4 (indicating inconsistency) in two maternal dimensions and in both child dimensions, while scores on the other dimensions resulted adequate (≥5). During T3 and T4 average scores resulted adequate (≥5) for all of the six dimensions. As far as it concerns the distribution of the dyads in the zones of the EA clinical screener, as it is possible to see in **Table 2** during the periods considered a progressively larger amount of dyads fell in the Emotionally Available (EA) zone, while the “lower” zones were progressively less represented.

**Table 1:** Average scores and standard deviations of the Emotional Availability Scales (EAS) applied during T1, T3, and T4.

	*M* (*SD*)		
Variables	T1 (*N* = 20)	T3 (*N* = 19)	T4 (*N* = 13)
*Sensitivity*	5.15 (1.07)	6.03 (1.06)	6.85 (0.38)
*Structuring*	4.93 (0.98)	5.76 (1.02)	6.73 (0.44)
*Non-intrusiveness*	4.73 (0.82)	5.79 (0.75)	6.54 (0.63)
*Non-hostility*	6.05 (0.94)	6.53 (0.79)	7.00 (0.00)
*Ch. Responsiveness*	4.68 (1.05)	5.58 (0.95)	6.50 (0.50)
*Ch. Involvement*	4.15 (1.09)	5.05 (1.04)	6.00 (0.79)

**Table 2:** Distribution of the dyads on the zones of the emotional availability (EA) clinical screener during T1, T3, and T4.

	T1 (*N* = 20)		T3 (*N* = 19)		T4 (*N* = 13)	
	Mother	Child	Mother	Child	Mother	Child
*EA Zone*	7 (35%)	5 (25%)	15 (78.95%)	14 (73.68%)	13 (100%)	13 (100%)
*Complicated Zone*	11 (55%)	10 (50%)	4 (21.05%)	5 (26.32%)	–	–
*Detached Zone*	2 (10%)	4 (20%)	–	–	–	–
*Problematic Zone*	–	1 (5%)	–	–	–	–

#### Psychological Distress

As far as it concerns psychological distress, Cronbach’s alpha was applied to the SCL-90-R symptom dimensions and to the global distress indexes. Good internal consistency was reported for obsessive-compulsive (0.82), interpersonal sensitivity (0.72), social phobia (0.72), paranoid ideation (0.78), psychoticism (0.80), and for the total of the items (0.90). Reliability was acceptable for somatization (0.67) and depression (0.70), poor for anxiety (0.56) and unacceptable for hostility (≤0.50), which was excluded from subsequent analysis. **Table 3** reports average scores, standard deviations and the distribution among normative cut-offs for the SCL-90-R scores. As it is possible to see, the majority of the subjects fell within the SCL-90-R normative cut-off values while a smaller percentage of mothers reported values above the norm, suggesting the presence of significant psychological distress.

**Table 3:** Average scores, standard deviations, and distribution of the mothers in the SCL-90-R.

*N* = 20		Norm	Clinical
	*M* (*SD*)	*N* (%)	*N* (%)
*Somatization*	746.40 (5.49)	18 (90)	2 (10)
*Obsessive-compulsive*	49.40 (9.52)	15 (75)	5 (25)
*Interpersonal-Sensitivity*	47.65 (6.95)	15 (75)	5 (25)
*Depression*	46.80 (5.19)	18 (90)	2 (10)
*Anxiety*	45.85 (4.83)	18 (90)	2 (10)
*Phobic*	45.95 (3.94)	19 (95)	1 (5)
*Paranoid Ideation*	46.50 (8.15)	17 (85)	3 (15)
*Psychoticism*	47.85 (7.57)	17 (85)	3 (15)
*GSI*	46.30 (5.60)	18 (90)	2 (10)
*PST*	45.48 (6.57)	19 (95)	1 (5)
*PSDI*	48.90 (6.69)	15 (75)	5 (25)

#### Couple Adjustment

Regarding couple adjustment, Cronbach’s alpha coefficient reported good internal consistency for the DAS total score (0.77) and for the subscales concerning Dyadic Consensus (0.69) and Dyadic Satisfaction (0.68). Reliability was unacceptable as far as it concerns Dyadic Cohesion and Affective Expression (α ≤ 0.50). Thus, these subscales were excluded from subsequent analysis. Normative cut-offs were computed from average scores and standard deviations reported in the article of the Italian validation of the DAS (Gentili et al., 2002). All the mothers in the present study reported scores that fell in the normative range (**Table 4**).

**Table 4:** Average scores, standard deviations, and distribution of the mothers’ scores in the DAS.

*N* = 18		<Norm	≥Norm
	*M* (*SD*)	*N* (%)	*N* (%)
*Dyadic Consensus (DC)*	54.39 (4.79)	–	18 (100)
*Dyadic Satisfaction (DS)*	42.44 (4.33)	–	18 (100)
*Dyadic Adjustment_TOT*	123.72 (9.47)	–	18 (100)

#### Perceived Social Support

The Cronbach’s alpha coefficient reported very good reliability for all the scales of the MSPSS, considering both the different subscales and the whole scale (0.76 ≤ α ≤ 0.97). **Table 5** reports average scores and standard deviations for the MSPSS. Normative cut-offs were extracted from the validation study by Prezza and Principato (2002). As it is possible to see, the majority of the subjects reported the perception of satisfactory support provided by family, friends and a significant other.

**Table 5:** Average scores, standard deviations, and distribution of the mothers in the MSPSS.

*N* = 20		≥Norm	<Norm
	*M* (*SD*)	*N* (%)	*N* (%)
*Support by a significant other*	5.43 (0.65)	19 (95)	1 (5)
*Support by family*	5.16 (1.18)	16 (80)	4 (20)
*Support by friends*	4.46 (1.06)	17 (85)	3 (15)
*Perceived social support_ TOT*	5.03 (0.81)	18 (90)	2 (10)

As it is possible to see from the data above, from a descriptive point of view at the beginning of the massage course all the mothers seemed to be able to rely on satisfactory couple adjustment, showing scores within the normative range in all the DAS scales. A similar consideration could be made for social support; although few subjects reported MSPSS scores below average, the majority of the participants seemed to experience a sufficient amount of support provided by family, friends and a significant other. As far as it concerns psychological wellbeing, although the majority of the subjects reported scores that fell into the SCL-90-R normative range, some mothers reported the presence of significant psychological distress as well. It is noteworthy, however, that rarely this perception reached the clinical cut-off. Finally, considering mother–child interactions, it is possible to see how, during T1, most of the mother–child dyads fell in the complicated zone of EA, indicating the presence of an emotional connection but the existence of difficulties as well. The complicated zone, and the zones below were progressively less represented during the ongoing of massage classes. During the follow up (T4), all the dyads that completed the program fell in the emotional available zone.

### Change vs. Stability in Quality of Mother–Child Interactions

To assess change vs. stability of EA during the infant-massage course the Friedman test was applied. This non-parametric statistical test can be considered a valid alternative of the parametric repeated measures ANOVA. The results reported a statistically significant increase in maternal sensitivity (*X*^2^ = 18.650, *p* = 0.001), structuring (*X*^2^= 17.190, *p* = 0.001), non-intrusiveness (*X*^2^ = 15.864, *p* = 0.001), and non-hostility (*X*^2^ = 7.400, *p* = 0.025), just as in child responsiveness (*X*^2^ = 15.650, *p* = 0.001) and involvement (*X*^2^ = 15.476, *p* = 0.001), indicating thus an improvement in all the EA dimensions during the massage course. To investigate the specific patterns of change in the scales, the Wilcoxon signed-rank test was applied to compare the different periods of infant massage course. This non-parametric statistical hypothesis test can be considered as an alternative to the paired Student’s *t*-test and can be used to compare repeated measures on a single sample to assess differences in the population mean ranks. The results reported a statistically significant increase in maternal sensitivity (*Z* = -2.553, *p* = 0.011), structuring (*Z* = -2.335, *p* = 0.020), non-intrusiveness (*Z* = -3.357, *p* = 0.001), just as in child responsiveness (*Z* = -2.626, *p* = 0.009), and involvement (*Z* = -2.120, *p* = 0.034) from T1 to T3. Regarding the transition from T3 to T4, statistically significant improvements were highlighted for maternal sensitivity (*Z* = -2.070), structuring (*Z* = -2.384, *p* = 0.017), non-intrusiveness (*Z* = -2.059, *p* = 0.040) and in child responsiveness (*Z* = -2.121, *p* = 0.034).

### Associations between Emotional Availability and Psychological Distress

To test for associations between quality of mother–child interactions and maternal psychological distress, the Spearman’s Rho coefficient was applied to the SCL-90-R reliable scores (obsessive-compulsive, interpersonal sensitivity, social phobia, paranoid ideation, psychoticism, somatization, depression, anxiety, GSI, PST, and PSDI) and to the scores obtained through the EAS during T1, T3, and T4. **Table 6** reports associations between EA and psychological distress. As it is possible to observe, regarding T1, correlations were found between maternal non-intrusiveness and psychoticicsm. During T3, negative correlations were found between psychoticism, maternal structuring, non-intrusiveness, non-hostility, and for child responsiveness. Moreover, anxiety resulted negatively associated with maternal non-intrusiveness and child responsiveness. As far it concerns T4, statistically significant inverse associations were found between maternal sensitivity and obsessive-compulsive, the GSI, and the PSDI.

**Table 6:** Associations between EA and psychological distress.

		*Som*	*OC*	*I-S*	*Dep*	*Anx*	*Phob*	*Par*	*Psy*	*GSI*	*PST*	*PSDI*
*Sens*	T1	-0.113	0.168	0.014	-0.198	-0.367	-0.197	-0.129	-0.134	-0.037	-0.029	-0.111
	T3	-0.123	0.227	-0.313	0.102	-0.201	-0.378	-0.108	-0.452	0.026	-0.118	0.127
	T4	-0.342	-0.628ˆ*	-0.401	-0.516	-0.468	0.139	-0.314	-0.301	-0.627ˆ*	-0.514	-0.628ˆ*
*Struct*	T1	0.058	-0.038	0.051	-0.029	-0.320	-0.098	0.020	-0.311	-0.061	-0.071	-0.050
	T3	-0.048	0.173	-0.369	-0.053	-0.323	-0.101	-0.111	-0.621ˆ**	-0.091	-0.233	0.079
	T4	0.007	-0.247	0.116	-0.211	-0.264	0.215	0.034	-0.029	-0.210	-0.034	-0.420
*Nonint*	T1	0.108	0.157	0.073	0.123	-0.249	-0.020	-0.038	-0.451ˆ*	0.102	-0.030	0.179
	T3	-0.222	0.053	-0.371	-0.240	-0.506ˆ*	0.088	-0.237	-0.591ˆ**	-0.252	-0.358	-0.084
	T4	0.027	-0.435	-0.234	-0.283	-0.130	0.463	-0.314	-0.175	-0.348	-0.247	-0.418
*Nonhos*	T1	0.060	0.035	0.177	-0.200	-0.201	-0.329	0.150	-0.189	-0.031	0.035	-0.129
	T3	-0.253	0.079	-0.413	-0.086	-0.337	-0.088	-0.280	-0.582ˆ**	-0.220	-0.392	0.047
	T4	–	–	–	–	–	–	–	–	–	–	–
*Ch. Resp*	T1	0.023	0.195	0.330	0.282	0.207	-0.061	0.237	0.050	0.248	0.302	0.086
	T3	-0.260	0.088	-0.352	-0.088	-0.495ˆ*	-0.281	-0.201	-0.599ˆ**	-0.152	-0.282	0.020
	T4	0.386	-0.407	-0.237	-0.215	-0.022	0.418	-0.451	-0.350	-0.257	-0.236	-0.257
*Ch. Involv*	T1	0.226	-0.051	0.145	0.139	-0.018	0.071	0.088	0.053	0.081	0.039	0.112
	T3	-0.127	0.242	0.030	0.089	-0.298	-0.510	0.047	-0.367	0.051	-0.015	0.016
	T4	0.222	-0.168	-0.046	0.029	-0.184	0.330	-0.334	-0.250	-0.125	-0.125	-0.086

*^∗^Correlation is significant at the 0.05 level (two-tailed). ^∗∗^Correlation is significant at the 0.01 level (two-tailed).*

### Associations between Emotional Availability and Couple Adjustment

To test for associations between quality of mother–child interactions and couple adjustment, the Spearman’s Rho coefficient was applied to the DAS reliable scores (Dyadic Consensus – DC, Dyadic Satisfaction – DS, and DAS total scores) and to the scores obtained through the EAS during T1, T3, and T4. **Table 7** reports associations between EA, couple adjustment and perceived social support. As it is possible to see, regarding T1, statistically significant inverse correlations were reported between maternal sensitivity, Dyadic Consensus and the DAS total score, and between maternal structuring and Dyadic Satisfaction. During T3, significant negative correlations were reported between maternal structuring, Dyadic Consensus and the DAS total score, and between maternal non-intrusiveness, Dyadic Consensus and the DAS total score. No statistically significant associations between Couple adjustment and EAS scores during T4 were found.

**Table 7:** Associations between EA, couple adjustment, and perceived social support.

		*DAS_DC*	*DAS_DS*	*DAS_Tot*	*MSPSS Other*	*MSPSS Family*	*MSPSS Friends*	*MSPSS*
*Sens*	T1	-0.578ˆ*	-0.306	-0.558ˆ*	0.027	0.207	-0.016	0.094
	T3	-0.443	-0.034	-0.387	-0.031	0.081	0.229	0.187
	T4	-0.543	-0.259	-0.402	0.059	0.321	-0.433	0.029
*Struct*	T1	-0.355	-0.477ˆ*	-0.449	-0.177	0.098	-0.183	-0.116
	T3	-0.539ˆ*	-0.171	-0.511	0.039	-0.045	-0.036	0.009
	T4	-0.112	-0.202	-0.027	0.087	0.446	-0.532	0.044
*Nonint*	T1	-0.270	-0.306	-0.250	0.122	0.364	0.079	0.238
	T3	-0.668ˆ**	-0.351	-0.630	0.113	-0.009	-0.103	-0.015
	T4	-0.255	-0.353	-0.182	0.231	0.178	-0.473	0.025
*Nonhos*	T1	-0.265	-0.359	-0.379	-0.473ˆ*	-0.214	-0.382	-0.460ˆ*
	T3	-0.291	-0.086	-0.313	0.185	0.056	0.112	0.102
	T4	–	–	–	–	–	–	–
*Ch. Resp*	T1	-0.211	-0.275	-0.255	-0.309	-0.054	0.092	-0.042
	T3	-0.378	-0.099	-0.374	0.088	0.049	0.086	0.049
	T4	0.107	-0.302	0.022	0.110	-0.066	-0.325	-0.097
*Ch. Involv*	T1	-0.354	-0.636ˆ**	-0.421	-0.110	0.033	0.088	0.029
	T2	-0.338	-0.153	-0.324	-0.001	0.049	0.288	0.092
	T3	0.039	-0.374	-0.062	0.459	0.464	-0.189	0.258

*^∗^Correlation is significant at the 0.05 level (two-tailed). ^∗∗^Correlation is significant at the 0.01 level (two-tailed).*

### Associations between Emotional Availability and Perceived Social Support

To test for associations between quality of mother–child interactions and the dimensions of perceived social support the Spearman’s Rho coefficient was applied to the scores of the MSPSS and to the EAS during T1, T3, and T4. As it is possible to observe in **Table 7**, regarding T1, negative associations were found between maternal non-hostility, the social support received by a significant other, and the total score of the MSPSS. No associations were found between EAS during T3 and T4 and the perceived social support.

### Associations between Psychological Distress, Couple Adjustment, and Perceived Social Support

Spearman’s Rho was applied to test for associations between perceived social support, couple adjustment, and psychological distress. No statistically significant associations were found between couple adjustment and perceived social support, neither between couple adjustment and psychological distress. With respect to psychological distress and perceived social support, statistically significant correlations were found between paranoid ideation and the support received by a significant other (*r* = -0.492, *p* = 0.027), the total score of the MSPSS (*r* = -0.512, *p* = 0.021).

### Associations between Improvements in Emotional Availability, Psychological Distress, Couple Adjustment, and Perceived Social Support

In order to assess whether there were associations between EA improvements among the three different timepoints and the other measures, the variance score of EA between timepoints (i.e., from T1 to T3, from T3 to T4, and from T1 to T4) was calculated subtracting the scores at later stages with scores at previous stages. Subsequently, Spearman’s Rho correlations were run between the variance scores of EA and the other measures (i.e., psychological distress, perceived dyadic satisfaction, and perceived social support). Some associations were detected for SCL-90-R, namely a negative relationship between higher psychological distress at three scales of the SCL-90-R (assessed at time 1) and lower variance scores between T1 and T4. More specifically, a negative relationship was detected between Interpersonal Sensitivity and variance score of Child Responsiveness (*r* = -537^∗^, *p* < 0.05), Social Phobia and variance score of Child Involvement (*r* = -519^∗^, *p* < 0.05), Psychoticism and variance score of Child Responsiveness (*r* = -531^∗^, *p* < 0.05). Taken together, these results indicate that the higher psychological distress mothers experience at T1, the less their interaction quality improved during massage lessons.

## Discussion

The first aim of the present study was to investigate EA during infant massage classes and to observe if an improvement in mother–child interactions occurred. Up to date, only few studies applied the EAS in the context of infant massage (Hays, 2014) and, at least to our knowledge, none to unselected populations. Secondly, according to the literature that highlights the intervention of multiple factors in determining the quality of parenting practices (Belsky, 1984; Feiring et al., 1987; Jennings et al., 1991; Melson et al., 1993; Bender and Losel, 1998; Singer et al., 2003; Favez et al., 2006), we aimed to test whether aspects such as the maternal perception of couple adjustment, social support and psychological wellbeing were associated to mother–child EA. At the beginning of the courses, the dyads showed scores that ranged from adequate to complicated EA. However, the results reported an increase in all the six dimensions concurrently with the ongoing of the infant massage course. Mothers enrolled in the courses seemed to gradually become more sensitive and responsive toward their children’s bids, as well as more able to provide adequate scaffolding during the lessons. At the same time, they seemed also to become less intrusive, providing more space to the interactions and being less interfering, both psychologically and physically, although the specific setting of infant massage explicitly requires tactile stimulation and the physical manipulation of the infant’s body. Simultaneously, a significant increase in child responsiveness was recorded, suggesting an improvement in the ability to find an adequate balance between self- and interactive-regulation and in the possibility to organize affects and behaviors in a coordinated way in order to respond to the caregiver’s bids. This parallel increase of maternal sensitivity and child responsiveness, the major dimensions indicative of the adult’s and the child’s EA, seems to support the hypothesis of bi-directionality and reciprocity within adult–child relationships (Sander, 1977; Tronick, 1989; Beebe and Lachmann, 2002). Although these improvements are not directly attributable to the course of infant massage, also due to the absence of a control group, this seems a plausible hypothesis, especially considering the literature concerning short-term stability of EA (Robinson et al., 1993; Biringen et al., 1995; Bornstein et al., 2006). Moreover, several studies report a positive influence on adult–child relationships exerted by infant massage (Onozawa et al., 2001; Lee, 2006). More research is needed in future in order to discern more clearly how these improvements in adult–child interactions might be affected by the massage course itself, by developmental processes or by the progressive mutual adjustment that the dyad reaches after delivery.

As far as it concerns maternal psychological wellbeing, as expected, a higher degree of adult psychopathology resulted associated with less optimal mother–child interactions, supporting the hypothesis that experiencing some kind of psychological distress might affect different domains of life, including the one of everyday interactions with one’s own child (Rogosch et al., 1992; Tronick and Weinberg, 1997; Anke, 2012). In particular, in our study, the major symptomatic scales negatively associated with the quality of mother–child exchanges were anxiety and psychoticism, considered as a graduated continuum from mild interpersonal alienation to first-rank symptoms of psychosis (Derogatis, 1977). Given the preliminary nature of this study, self-report measures were administered only during the first period considered; it would be interesting, in the future, to administer them also at the end of the massage course, in order to see whether an improvement in maternal psychological wellbeing occurs parallel to the improvement of mother–child interactions.

Our hypotheses were not confirmed as far as it concerns the associations between mother–child interactions, couple adjustment and social support. The lack of associations seems to reflect in part the non-univocal results reported by the literature. In fact, although several studies reported the presence of associations between marital quality and parenting (Erel and Burman, 1995) the nature of these associations was not always clear (Grych, 2002). Sometimes they appeared positive (Carneiro et al., 2006), sometimes they were negative (Favez et al., 2006), in other cases they were absent (Barnett et al., 2008; Favez et al., 2013). This incongruence could be due to many methodological reasons, such as different samples, the different periods when the measures were administered as well to the use of different kinds of measures (observational vs. self-report). Moreover it is possible that the “spill over” effect (Engfer, 1988; Katz and Gottman, 1996) expected from marital quality and perceived social support toward adult–child interactions might differ according to the family system investigated (i.e., parental-dyadic vs. co-parental-dyadic) (McHale et al., 2000; Johnson, 2001; Lindsey and Caldera, 2006; Stroud et al., 2015). It is important to note that, differently from what it is often reported in the literature, all our mothers held satisfactory couple relationships. Maybe they were still experiencing the so-called “baby honeymoon” (Hobbs, 1965; Wallace and Gotlib, 1990) or maybe they were part of that portion of couples that do not face a decline in marital satisfaction after delivery (Holmes et al., 2013). Anyway, further analysis will be required in future, also expanding and improving research designs, in order to confirm or disconfirm these associations and to examine in depth their nature.

The present study shows a series of limitations that might offer useful suggestions for future research. The first limit regards the sample; the small amount of the participants and the absence of a non-treated control group, in fact, prevent us to generalize the obtained results. A larger sample would allow to adopt more sound statistical analysis, while the presence of a control group would allow to compare the development of mother–child interactions between dyads that undergo infant massage courses and dyads without intervention, thus leading to a better explanation of the effective influence of infant massage upon the establishment of early adult–child interactions.

Another limit regards the absence of a baseline assessment of mother–child interactions. Videotaping the dyads during massage lessons might have influenced the nature of mother–child interactions both in positive or in negative. On one side, in fact, the massage context might have acted as a buffering factor, preventing the mothers from enacting dysfunctional behaviors that otherwise could have been adopted; on the other hand, instead, finding themselves in a new situation and being asked to do something new (massaging their babies while being videotaped) might have made interactions more challenging for these women. In this sense, including a baseline assessment in future would favor a better control of the different intervening variables. Moreover, it should be taken into account that our study only included mothers and did not involve fathers. Expanding the research design in this direction in future would lead to two major consequences: first of all, the possibility to support and sustain also paternal functioning during the postpartum period; secondly the opportunity to increase and to deepen the comprehension of family processes.

Finally, some considerations should be dedicated to the clinical implications of our study. These preliminary findings, in fact, seem to suggest the usefulness of infant massage for the strengthening and the enhancement of early healthy adult–child interactions. This cost-saving technique could provide a simple but effective way to favor the construction of early affective bonds; in this way, it could accompany a process existing per se and sustain the dyad during expected developmental challenges, whether necessary. Especially in a delicate interval such as the post-partum period this practice could become extremely important, since it could help the dyad to face the need of mutual adjustment, facilitating regulatory processes and the establishment of sleep-wake cycles. Moreover, a “guided” emotionally intense approach toward the infant could reassure the mothers, who often perceive the newborns as fragile and are afraid to touch them, making them more confident when handling their babies. From this perspective, infant massage constitutes a precious resource in terms of primary prevention, i.e., in terms of those interventions aimed at sustaining and enhancing the existing resources within the family system, since it can be offered as an enriching support also in the absence of adult psychopathology. A replication of these results in larger samples would thus encourage the diffusion of this non-invasive technique in terms both of relational support and enhancement of parenting abilities.

## Ethics Statement

The study was carried out in accordance with the recommendations of the Code of Ethics approved by the General Assembly of the Italian Association of Psychology held on March 27, 2015. Written consent was obtained from the participants.

## Author Contributions

AS prepared the study design and supervised the research team; GB carried out the massage courses and recruited the sample. SF collected data and prepared data set. AP and MP wrote the introduction section of the manuscript, performed statistical analyses, and prepared tables and figures. AS, MP, and AP wrote the discussions section of the manuscript. All authors reviewed the manuscript.

## Conflict of Interest Statement

The authors declare that the research was conducted in the absence of any commercial or financial relationships that could be construed as a potential conflict of interest.

## Footnotes

1 This study was carried out in accordance with the recommendations of the Code of Ethics approved by the General Assembly of the Italian Association of Psychology held on March 27, 2015 with written informed consent from all subjects.
